# Precision treatment of chalazion based on AI-driven risk stratification: a prospective study of triamcinolone acetonide combined with intense pulsed light therapy

**DOI:** 10.3389/fmed.2026.1822984

**Published:** 2026-06-15

**Authors:** Yanlin Zhong, Yiming Hu, Yuan Lin, Huping Wu

**Affiliations:** 1 School of Medicine, Xiamen Eye Center and Eye Institute of Xiamen University, Xiamen, China; 2 Xiamen Clinical Research Center for Eye Diseases, Xiamen, Fujian, China; 3 Xiamen Key Laboratory of Ophthalmology, Xiamen, Fujian, China; 4 Fujian Key Laboratory of Corneal and Ocular Surface Diseases, Xiamen, Fujian, China; 5 Xiamen Key Laboratory of Corneal and Ocular Surface Diseases, Xiamen, Fujian, China; 6 Translational Medicine Institute of Xiamen Eye Center of Xiamen University, Xiamen, Fujian, China; 7 Ophthalmic Center, The Second Affiliated Hospital, Jiangxi Medical College, Nanchang University, Nanchang, China

**Keywords:** artificial intelligence, chalazion, recurrence prediction, risk stratification, XGBoost

## Abstract

**Purpose:**

To evaluate the rates of chalazion recurrence following intralesional triamcinolone acetonide (TA), intense pulsed light (IPL), or combined TA + IPL therapy and to validate an AI-enhanced risk stratification strategy.

**Methods:**

This prospective, observational study enrolled 366 patients. To eliminate immortal time bias and circular reasoning, LASSO-Cox regression with 10-fold cross-validation was employed to identify purely baseline predictors: age, Demodex infection, chronicity, and meibomian gland dysfunction (MGD). These baseline predictors were integrated into a clinical Cox proportional hazards model and a survival-based XGBoost model, which were subsequently merged into a hybrid framework. Performance was assessed via time-dependent area under the curve (AUC), net reclassification improvement (NRI), Kaplan–Meier analysis, and decision curve analysis (DCA).

**Results:**

The 3-month complete resolution rate was significantly higher in the combined TA + IPL group (95.2%) compared to the TA-only (90.8%) and IPL-only (79.4%) groups (*p* < 0.001). During follow-up, 60 recurrence events (16.4%) occurred. Recurrence rates differed significantly (χ^2^ = 31.73, *p* < 0.001) as follows: IPL group (38.2%), TA group (15.4%), and TA + IPL group (8.3%). Multivariate Cox analysis revealed that demodex infection, age, chronicity, and MGD were independent risk factors for chalazion recurrence. The hybrid model achieved an AUC of 0.822, outperforming the clinical (0.745) and XGBoost (0.821) (*p* < 0.05) models. AI integration significantly refined the risk estimates (NRI = 0.365, *p* = < 0.001). In the high-risk subgroup, recurrence rates reached 83.3–91.7% with monotherapy, whereas combined TA + IPL therapy significantly reduced the recurrence rate to 60.0%. DCA confirmed that the hybrid model consistently yielded the greatest net clinical benefit.

**Conclusion:**

A hybrid AI-clinical model significantly improved the prediction of chalazion recurrence. Combined TA + IPL therapy demonstrated superior efficacy, particularly for high-risk patients. This framework provides a robust decision-support tool for precision management and the systematic reduction of chalazion recurrence.

## Introduction

Chalazion represents a localized granulomatous inflammatory response triggered by the obstruction of meibomian gland ducts, which leads to lipid retention and subsequent extravasation into the tarsal collagenous stroma (1, 2). Although chalazion is frequently classified as a benign and self-limiting condition, it remains among the most prevalent eyelid disorders in clinical practice, often causing considerable ocular discomfort, visual disturbances, esthetic concerns, and a substantial psychological burden in patients (3). Notably, the incidence of chalazion is considerably greater in children than in adults, potentially because of meibomian gland secretory functions are more robust and proliferative capacity is increased in the pediatric population (4, 5). Compared with adult patients, pediatric patients present distinct clinical challenges, including a higher frequency of multiple lesions, greater susceptibility to secondary infection, and a markedly increased recurrence rate (6). Beyond its mere cosmetic impact, chalazion can precipitate various ocular surfaces and cause visual complications, such as meibomian gland loss (7), compromised tear film stability (8), induced astigmatism (9), and increased high-order aberrations (10). For children in the critical window of vision development, persistent chalazion or complications such as ptosis and conjunctival scarring from repeated surgeries may even lead to irreversible amblyopia (11).

During disease progression, the continuous accumulation of lipids secreted by meibomian cells stimulates a localized immune cascade, which manifests as masses on the eyelid and functional visual impairment in severe cases (12). Despite the clinical ubiquity of chalazion across all age groups, its underlying pathogenesis has yet to be fully elucidated (13). In a lipidomics-based study, ultra-performance liquid chromatography–mass spectrometry (UPLC–MS) was utilized to systematically analyze specimens collected from 2 to 28 weeks. The findings indicated that as disease progresses, the lipid composition within the cyst shifts from a near-normal profile to a state of severe lipid dysregulation. Specifically, a rapid surge in free cholesterol accompanied by the replacement of very-long-chain lipids with a mixture of short-chain esters, triacylglycerols, ceramides, and sphingomyelins alters the physical properties of meibum, facilitating its abnormal accumulation. This biochemical transformation is thought to be a fundamental driver of persistent chronic inflammation (14).

Ocular surface dysbiosis may also play a pivotal role in the evolution of chalazion. While viruses, *Demodex*, and bacterial blepharitis have not been definitively confirmed as direct etiologic agents of chalazion, the frequent history of recurrence and efficacy of antimicrobial therapy in certain patients suggest bacterial involvement. The detection of *Cutibacterium acnes* (*C. acnes*) PAB antibodies in excised samples further highlights the potential role of bacteria in disease development (15). Moreover, patients with chronic blepharoconjunctivitis or meibomian gland dysfunction (MGD) exhibit significantly higher incidence and recurrence rates, as chronic inflammatory stimuli lead to structural gland loss and poor secretion quality, creating a favorable environment for recurrent ductal obstruction (16).

Current management strategies for chalazion include traditional warm compresses, meibomian gland expression (MGX), and surgical excision (17). However, these conventional interventions often struggle to mitigate the high recurrence rate of chalazion, leading to prolonged impacts on patient well-being (18). In recurrent cases, repeated surgical trauma can result in conjunctival-tarsal scarring, ductal damage, or proximal gland obstruction, paradoxically triggering further granuloma formation (19). Other complications include madarosis, eyelid margin deformity, and secondary dry eye symptoms (20). Thus, exploring safer, more effective, and noninvasive therapeutic strategies based on a deeper understanding of the pathogenesis of chalazion is of paramount clinical importance.

Intense pulsed light (IPL), a broad-spectrum, noncoherent light source generated by a high-output xenon lamp, has evolved rapidly since its inception in 1994 (21). The ophthalmic application of IPL was pioneered in 2002 after observations that IPL therapy for rosacea significantly improved concurrent MGD and dry eye symptoms (22). Subsequent research has validated the safety and efficacy of IPL in managing MGD, particularly when IPL is combined with MGX, by enhancing meibum quality, improving discharge, and prolonging the tear break-up time (TBUT) (23). Proposed mechanisms underlying these effects include liquefying abnormal meibum via photothermal effects, reducing the microbial load (such as *C. acnes* and *Demodex*), and modulating local inflammatory mediators (24). Emerging evidence suggests that IPL plus MGX can reduce lesion volume and improve eyelid margin health, offering a safe and effective alternative for the treatment of multiple recurrent chalazia that might otherwise require surgery (25). Studies have demonstrated that IPL combined with meibomian gland massage can safely and effectively treat multiple recurrent meibomian cysts without surgical intervention or curettage by reducing cyst volume, improving eyelid margin abnormalities, and decreasing the grade of meibomian gland secretions (26).

Despite growing interest, standardized selection criteria for IPL as an adjunctive therapy remain poorly defined. Chalazion exhibits significant heterogeneity in terms of lesion characteristics, MGD severity, and recurrence risk. A “one-size-fits-all” approach may lead to the overtreatment of low-risk patients but undertreatment of high-risk individuals. Consequently, we hypothesize that IPL may exert therapeutic effects by regulating the ocular surface microbiota and restoring gland function. This prospective, real-world study aims to compare the efficacy of intralesional triamcinolone acetonide (TA) injection, standalone IPL, and combined TA + IPL therapy. Importantly, we seek to develop and validate a recurrence prediction model that integrates clinical variables with AI-derived probability assessments, facilitating a precision medicine approach to optimize clinical outcomes while minimizing unnecessary interventions.

## Method

### Study design and ethics approval

This prospective, observational study was conducted at the Xiamen Eye Center of Xiamen University. The protocols applied in this study adhered to the tenets of the Declaration of Helsinki and received formal approval from the Institutional Review Board of the Xiamen Eye Center. Prior to enrollment, written informed consent was obtained from all participants or their legal guardians.

### Participants

Patients who were consecutively diagnosed with chalazion between January 2024 and February 2025 were screened for inclusion. A diagnosis of chalazion was established by senior ophthalmologists via slit-lamp biomicroscopy and comprehensive clinical evaluation. The inclusion criteria were as follows: (1) clinically confirmed chalazion, (2) age between 1 and 82 years, and (3) commitment to the scheduled follow-up regimen. The exclusion criteria were as follows: (1) acute infectious hordeolum, (2) prior surgical intervention on the index lesion, (3) systemic corticosteroid or immunosuppressive therapy within the preceding month, and (4) concurrent ocular surface infections or severe eyelid pathologies.

### Treatment modalities

Patients were stratified into three treatment cohorts on the basis of the intervention received: intralesional TA injection, IPL therapy, or combined TA + IPL therapy. Treatment allocation resulted from shared clinical decision-making, accounting for lesion morphology, ocular surface status, and patient preference. For the TA cohort, triamcinolone acetonide was administered directly into the lesion under sterile conditions; a second injection was permitted after 2–4 weeks for partial responders. IPL sessions were performed using the M22 system (Lumenis Ltd., Yokneam, Israel) at 3- to 4-week intervals for a standard course of three sessions, with adjustments made on the basis of clinical response. All participants received standardized lid hygiene instructions throughout the study.

### Clinical assessment and follow-up

Baseline documentation included demographic information, lesion characteristics (size, quantity, anatomical location, and duration), and ocular surface parameters. Meibomian gland function was quantified using a standardized grading system. Follow-up assessments were performed at 1, 3, and 6 months post-intervention. At each visit, lesion status, ocular surface metrics, and any adverse events were recorded. Recurrence monitoring was extended to 12 months.

## Outcome measures

The primary endpoint was the complete resolution rate evaluated at 3 months post-treatment, defined as the total disappearance of the palpable chalazion nodule and localized chronic inflammation upon clinical examination.

The secondary endpoint was the recurrence-free survival (RFS) rate over a 12-month longitudinal follow-up period. To address the clinically multiple and bilateral nature of chalazia, recurrence was operationally defined as the development of any new chalazion lesion or the reappearance of the initial lesion within the same treated eye (study eye) after complete clinical resolution had been confirmed at the 3-month landmark. Lesions occurring in the contralateral eye were recorded separately and excluded from the primary recurrence analysis to avoid statistical confounding.

### Development of a recurrence prediction model

To identify robust predictors while minimizing overfitting, least absolute shrinkage and selection operator (LASSO)-Cox regression with 10-fold cross-validation was employed. Variables with nonzero coefficients at the optimal lambda were selected for multivariate modeling. A multivariate Cox regression model was subsequently constructed to estimate recurrence probability, from which a predictive nomogram was developed.

For enhanced clinical utility, a simplified point-based scoring system was derived by normalizing the regression coefficients. Simultaneously, an eXtreme Gradient Boosting (XGBoost) model was developed using the same predictors, with hyperparameters optimized via internal cross-validation. Finally, a hybrid framework was established by merging the AI-derived probabilities with the clinical scoring system using Cox regression.

### Evaluation of model performance

Model discriminative power was quantified using time-dependent the area under the curve (AUC) from receiver operating characteristic (ROC) analysis, with comparisons performed via the DeLong test. Calibration was evaluated through bootstrapping (1,000 iterations) and visualized with calibration plots, complemented by the Hosmer–Lemeshow goodness-of-fit test. The net reclassification improvement (NRI) was calculated at preset risk thresholds (0.2 and 0.5) to assess the added value of AI integration. Clinical utility was evaluated using decision curve analysis (DCA). For time-to-event outcomes, Kaplan–Meier curves and log-rank tests were used, with hazard ratios (HRs) estimated via multivariate Cox proportional hazards regression. Model interpretability was explored through SHapley Additive exPlanations (SHAP) values to quantify feature contributions to the predicted risk.

### Statistical analysis

For longitudinal RFS analysis, time-to-event was calculated from the date of initial treatment. Non-responders, defined as patients who failed to achieve complete resolution at the 3-month primary endpoint, were statistically handled as having an event occurrence at time 0 (0 months of recurrence-free survival). These patients were considered treatment failures from the baseline and were appropriately locked into the event matrix, ensuring they never incorrectly entered the true “at-risk” pool for subsequent post-resolution recurrence. Continuous variables are presented as the mean ± standard deviation or median (interquartile range) as appropriate. Categorical data are summarized as frequencies and percentages. Intergroup comparisons were performed by Student’s *t* test, the Mann–Whitney U test, the chi-square test, or Fisher’s exact test according to the data distribution. Statistical analyses were performed using R (version 2026.01.0; R Foundation for Statistical Computing). All tests were two-sided, with *p* < 0.05 considered to indicate statistical significance.

## Results

### Baseline characteristics and clinical outcomes

The initial cohort consisted of 380 cases. After excluding 14 cases (3.7%) lost to follow-up, 366 cases remained for analysis. The final analysis included 366 patients. No statistically significant differences in terms of baseline age, sex distribution, or lesion anatomical location were observed across treatment cohorts (all *p* > 0.05). However, the prevalence of moderate-to-severe MGD was higher in the combined therapy group. The median time to lesion resolution was 14.1 days for the TA-only group, 32.5 days for the IPL-only group, and 7.6 days for the combined TA + IPL group. At the 3-month post-treatment landmark (the primary endpoint), the complete clinical resolution rates differed significantly (χ^2^ = 19.82, *p* < 0.001): complete resolution was achieved in 90.8% (118/130) of the TA-only group, 79.4% (54/68) of the IPL-only group, and 95.2% (160/168) of the combined TA + IPL group.

During the study, 60 patients (16.4%) experienced recurrence. This included 34 non-responders who failed to resolve at 3 months (and were strictly modeled as having an event occurrence at time 0: 12 in the TA group, 14 in the IPL group, and 8 in the combined group) plus 26 patients who experienced a secondary true recurrence within the same treated eye after initial resolution. Recurrence rates varied significantly across modalities (χ^2^ = 31.73; *p* < 0.001): the IPL-only group experienced the highest recurrence (38.2%; 26/68), followed by the TA-only group (15.4%; 20/130), while the combined TA + IPL group demonstrated the most favorable outcome (8.3%; 14/168). In the TA group, the incidence of localized tissue atrophy was 2.1%. In the IPL group, erythema or pigmentation occurred in 4.9% of cases. All adverse events were mild and self-limiting.

### Predictor selection and multivariate modeling

**Figure 1:**
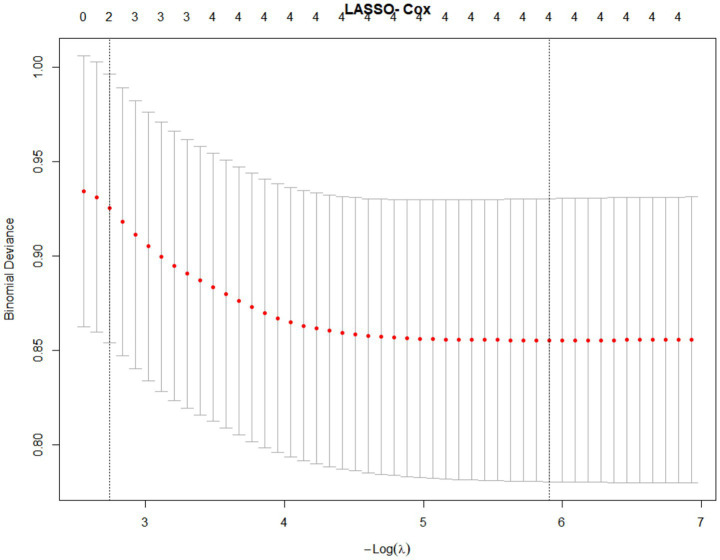
Variable selection via LASSO-Cox regression. The plot illustrates the optimization of model parameters through 10-fold cross validation. Red dots represent binomial deviance across different parameter values. The two dashed lines indicate the parameters that correspond to the minimum error and the most parsimonious model (one standard error rule). The numbers at the top represent the count of features retained in the model as the penalty parameter varied.

To establish a robust predictive framework, LASSO regression analysis with 10-fold cross-validation was utilized to minimize overfitting. On the basis of the minimum cross-validation error, six variables with nonzero coefficients were retained: age, treatment modality, intervention frequency, demodex infection, chronicity, and MGD (Figure 1).

**Figure 2:**
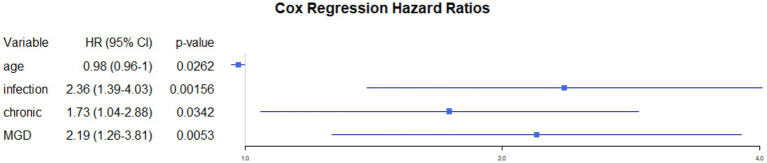
Multivariate cox proportional hazards analysis of recurrence predictors. The forest plot displays hazard ratios (HRs) and 95% confidence intervals (CIs) derived from the multivariate Cox regression. Age was inversely correlated with recurrence (HR 0.98, *p* = 0.0262). chronicity (HR 1.73, *p* = 0.0342), demodex infection (HR 2.36, *p* = 0.0015), and meibomian gland dysfunction (MGD) (HR 2.19, *p* = 0.0053) emerged as significant independent predictors. Squares represent point estimates, and horizontal lines denote 95% CIs.

Subsequent multivariate Cox proportional hazards modeling confirmed that these predictors were independently associated with recurrence risk. The forest plot in Figure 2 shows the hazard ratios (HRs) and 95% confidence intervals (CIs). Notably, chronicity (HR 1.73, *p* = 0.0342), demodex infection (HR 2.36, *p* = 0.0015), and MGD (HR 2.19, *p* = 0.0053) were identified as major risk factors.

### Model integration and diagnostic performance

**Figure 3:**
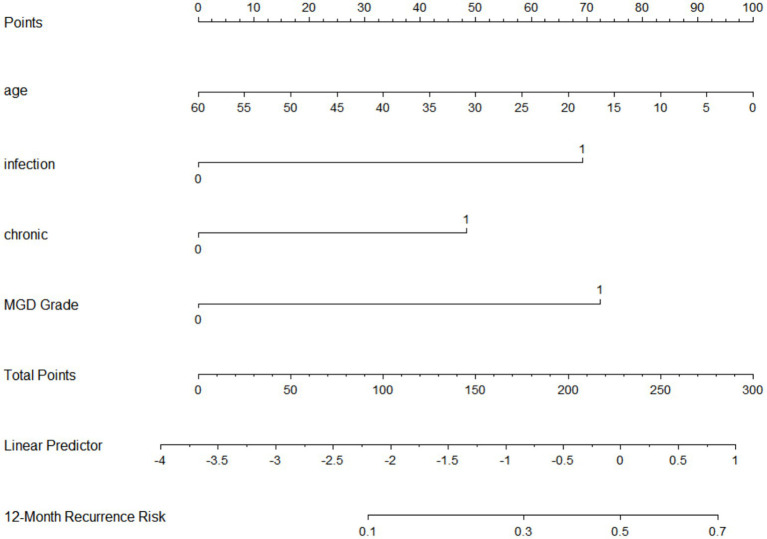
Nomogram for predicting individualized recurrence risk in patients with chalazion. This predictive tool integrates clinical and demographic factors—age, infection status, chronicity, demodex infection and MGD—to estimate individualized recurrence probabilities. To use the nomogram, locate the patient’s value on each variable axis, and draw a vertical line upward to determine the assigned points. The sum of these points is located on the “Total Points” axis, which corresponds to the “Linear Predictor” and the specific “Risk of Recurrence” scale at the bottom.

Variables that did not reach statistical significance in the Cox model (e.g., treatment frequency, *Demodex* infection) were nonetheless retained as covariates in the final nomogram and hybrid model to ensure informational integrity and robustness. The resulting nomogram provided individualized risk assessments by assigning weighted scores to each predictor (Figure 3). For clinical ease of use, a point-based scoring system was developed.

**Figure 4:**
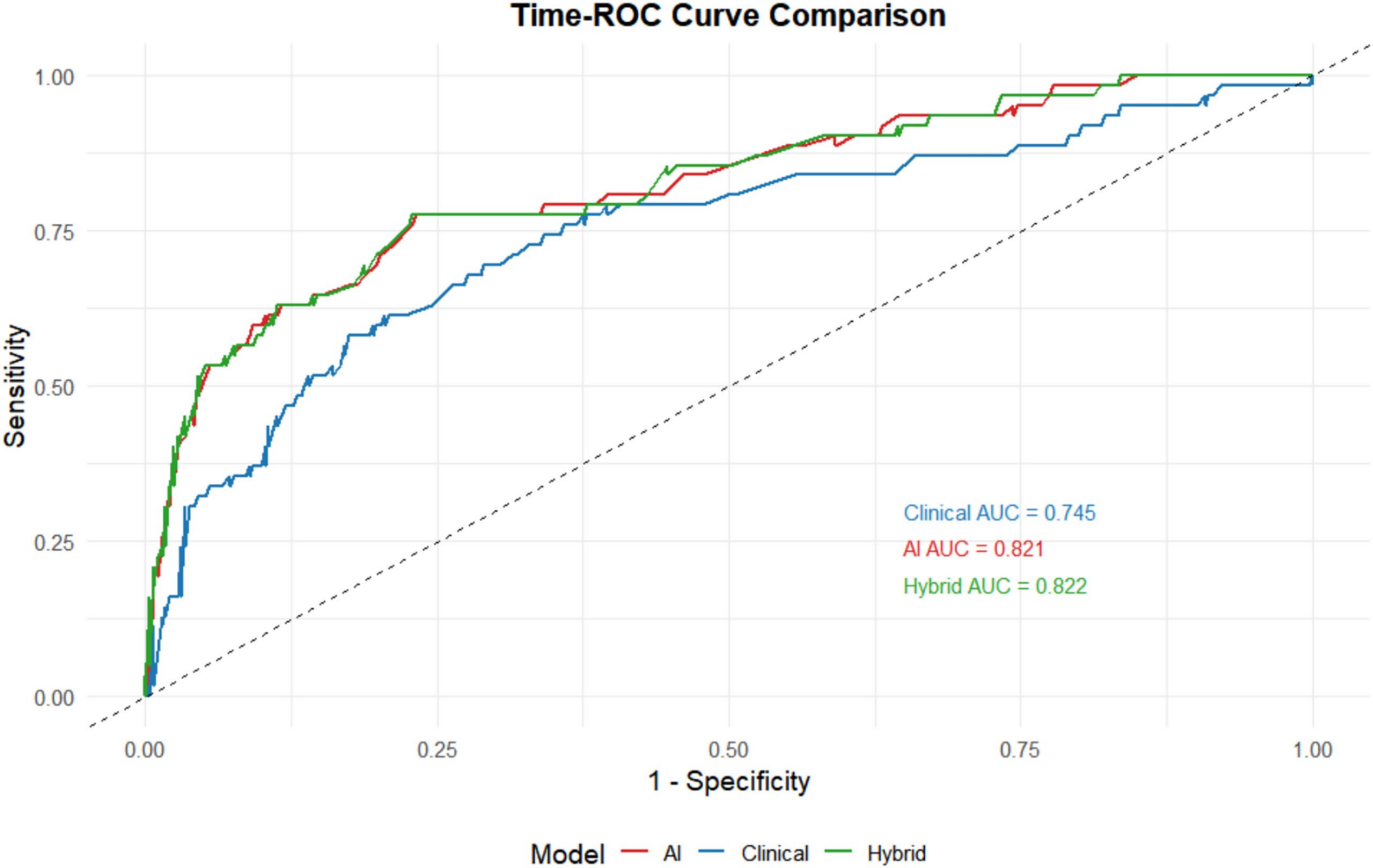
Comparison of time-ROC curves among clinical, AI, and hybrid models. Receiver operating characteristic (ROC) curves illustrate the diagnostic performance of the clinical model (AUC = 0.745), the XGBoost-based AI model (AUC = 0.821), and the hybrid model (AUC = 0.822). The hybrid framework, which integrates clinical variables with AI-derived probabilities, demonstrates the highest discriminative power. The diagonal dashed line represents the line of identity (AUC = 0.5). The incremental improvement in the AUC supports the added value of merging machine learning with traditional clinical predictors.

The clinical-only model yielded an AUC of 0.745. In comparison, the XGBoost-based AI model achieved an AUC of 0.821. The integration of AI-derived probabilities with clinical scores—forming the hybrid model—reached a superior AUC of 0.822 (Figure 4). DeLong tests confirmed that the hybrid model significantly outperformed both the standalone clinical and AI models (*p* < 0.05).

### Model validation and calibration

**Figure 5:**
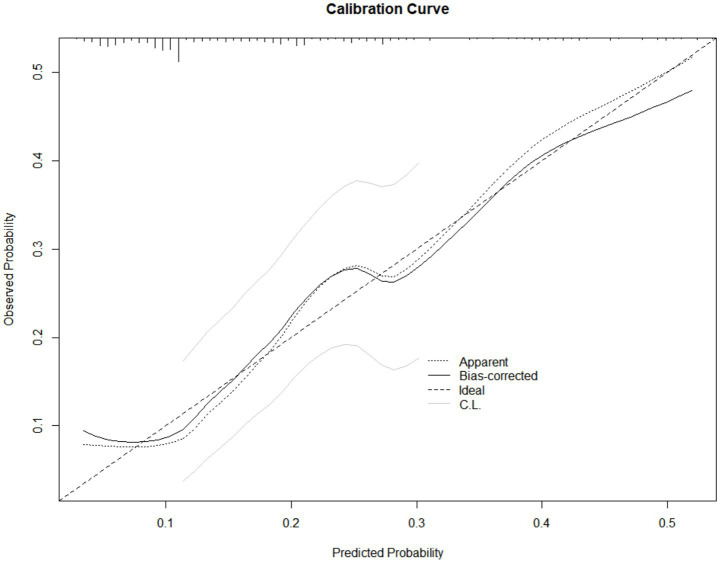
Calibration curve for the hybrid model. This plot assesses the agreement between the predicted and observed recurrence probabilities. The dashed diagonal line represents ideal model performance, whereas the solid line indicates the bias-corrected performance of the hybrid model. Close alignment between the solid line and the diagonal line indicates high predictive accuracy and minimal calibration error.

Internal validation of the hybrid model was performed via 1,000 bootstrap iterations. The calibration curve (Figure 5) demonstrated high concordance between the bias-corrected predicted probabilities and observed outcomes, with the curve closely aligning with the 45° ideal reference line. Key metrics further substantiated the reliability of the model: the corrected C-index was 0.813, the Brier score was 0.1012, and the calibration slope was 0.968, indicating exceptional stability and a lack of significant over- or underfitting.

### Reclassification and clinical utility

**Figure 6:**
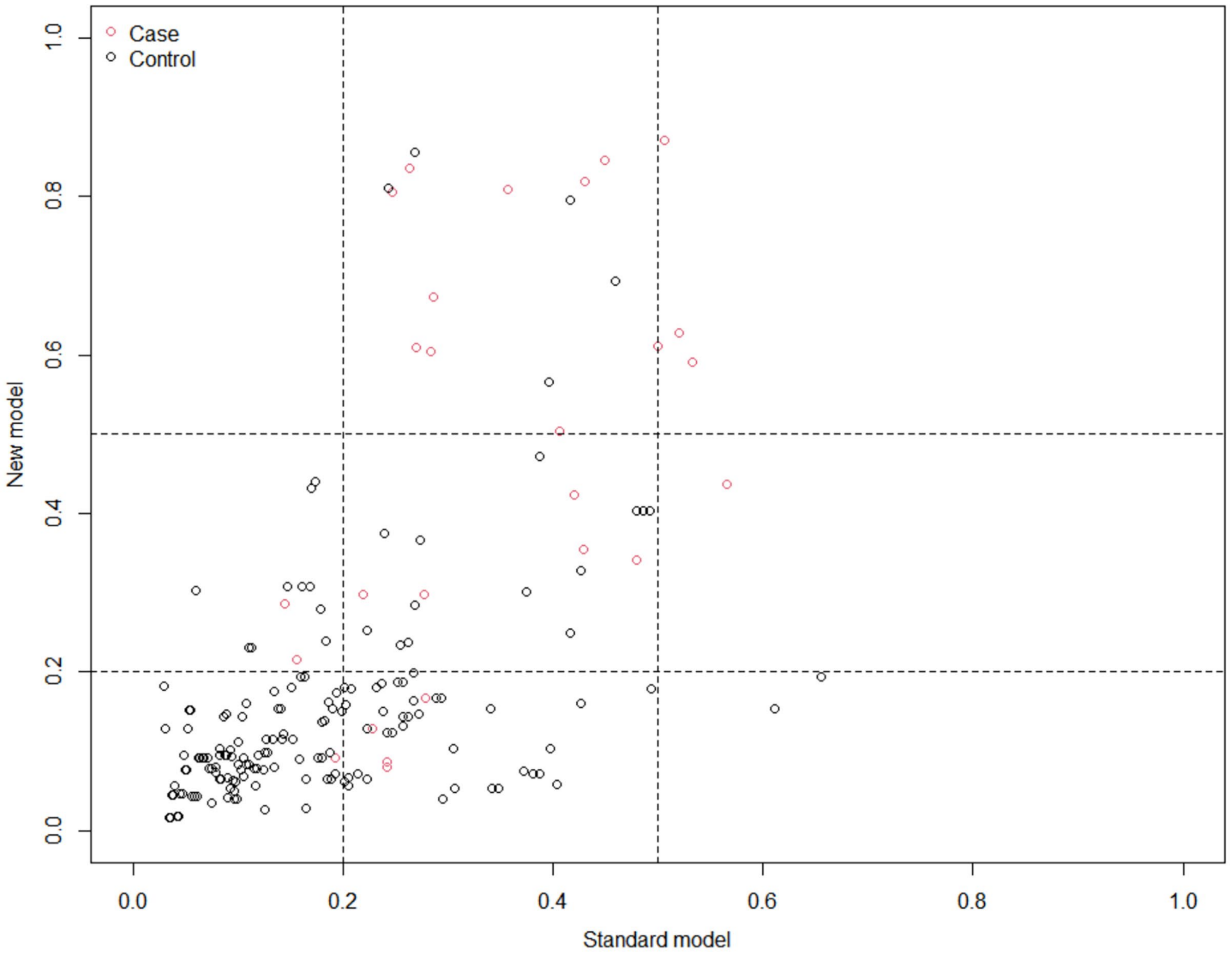
Net reclassification improvement (NRI) analysis. The scatter plot compares the predicted recurrence probabilities obtained with the standard clinical model (x-axis) and the AI-enhanced model (y-axis). Dashed lines denote preset risk thresholds (0.2 and 0.5), which were used to categorize patients into low-, medium-, and high-risk groups. Red circles represent cases of recurrence, and black circles represent nonrecurrence controls. The shifting of points above the diagonal for cases and below the diagonal for controls indicates refined risk classification by the AI-enhanced model.

**Figure 7:**
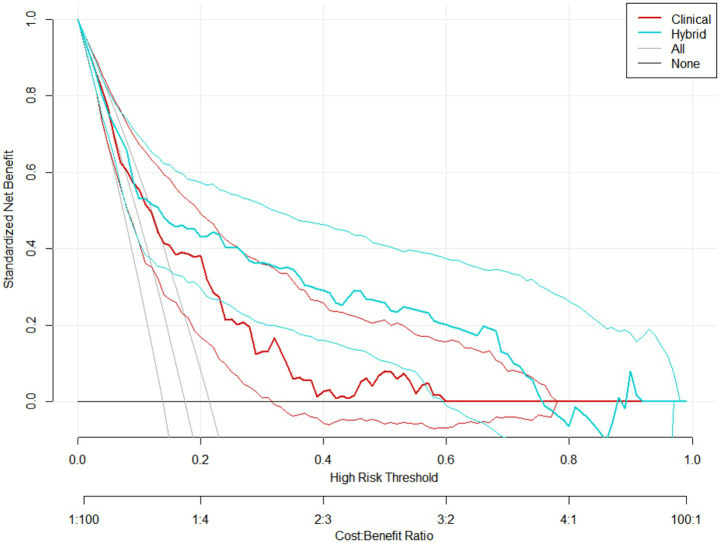
Decision curve analysis (DCA) comparing the clinical and hybrid models. The decision curve illustrates the standardized net benefit across various high-risk threshold probabilities. The AI-enhanced hybrid model consistently outperforms the clinical model. The “Treat All” and “Treat None” lines represent the treatment of all patients or none, respectively. The superior performance of the hybrid model across clinically relevant thresholds supports its utility in guiding recurrence prevention strategies.

Net reclassification improvement (NRI) analysis was conducted to evaluate the added value of AI integration. At risk thresholds of 0.2 and 0.5, the AI-enhanced model significantly refined risk categorization, achieving a total NRI of 0.365 (95% CI 0.183–0.552, *P* ≤ 0.001). This improvement was driven primarily by the accurate upward reclassification of recurrence cases (NRI+ = 0.225) (Figure 6). Decision curve analysis (DCA) further corroborated the hybrid model’s utility, showing superior standardized net benefits across the clinical threshold range of 0.10 to 0.60 compared with the “treat-all” or “treat-none” strategies (Figure 7).

### Risk stratification and interpretability

**Figure 8:**
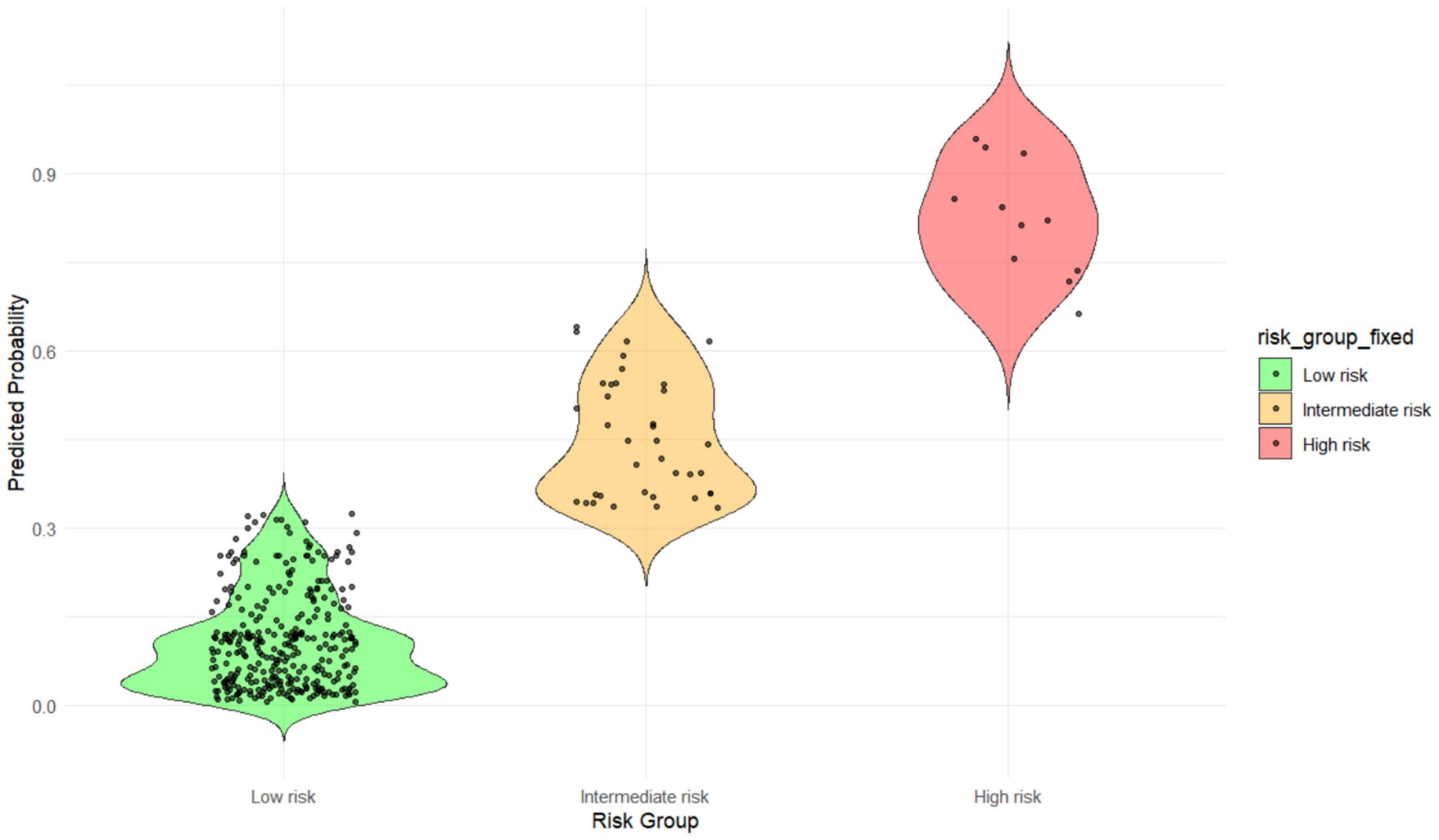
Distribution of predicted probabilities across risk strata. Violin plots validate the ability of the model to differentiate between risk populations. The subjects were categorized into low-, medium-, and high-risk groups on the basis of the predicted probabilities of recurrence (y-axis). The width of each violin represents the density of the data distribution, demonstrating a clear gradient in the predicted risk across the identified strata.

**Figure 9:**
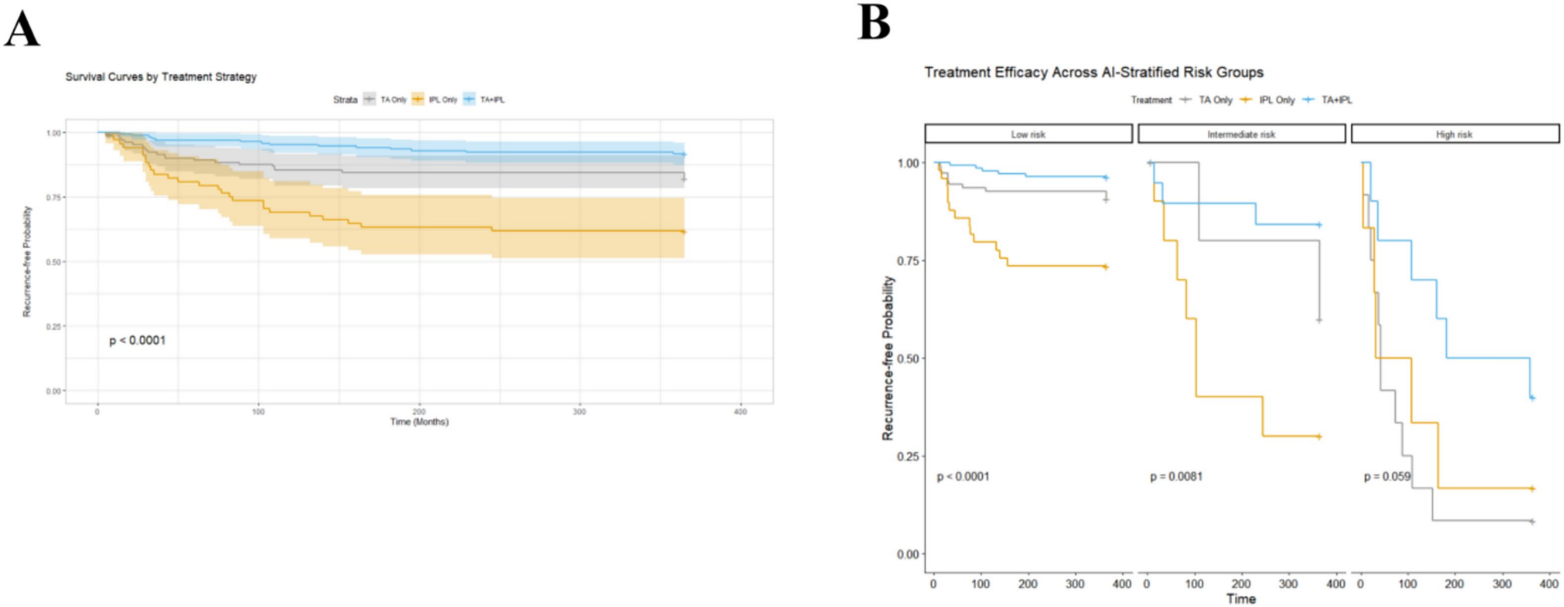
Kaplan–Meier curves of recurrence-free survival (RFS) by treatment and risk stratification. **(A)** Overall comparison: The TA + IPL group (blue) showed superior RFS compared to TA-only and IRI-only cohorts (*p* < 0.0001). Shaded areas indicate 95% CIs. **(B)** AI-stratified subgroups: TA + IPL consistently yielded the highest RFS in low- and intermediate-risk groups. A similar clinical trend was observed in the high-risk group, although not statistically significant (*p* = 0.059).

**Figure 10:**
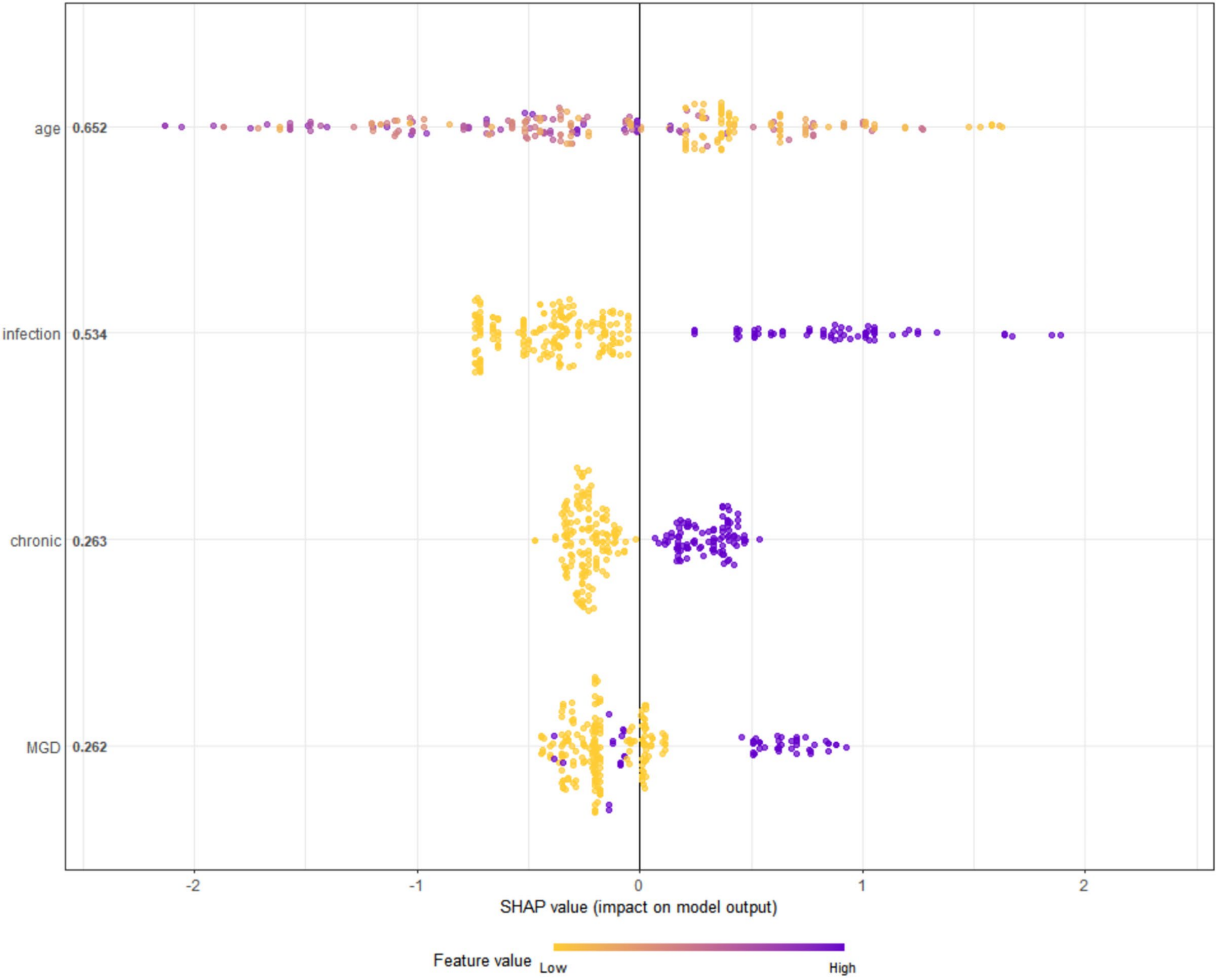
SHAP summary plot for feature interpretability. The bee-swarm plot displays the distribution of SHapley Additive exPlanations (SHAP) values for each feature, ranked by their global importance (mean absolute SHAP value). Each dot represents an individual patient. The color gradient indicates the feature value (yellow: low; purple: high). The horizontal position indicates whether a feature increases (positive SHAP) or decreases (negative SHAP) the predicted probability of recurrence.

Patients were stratified into low-, medium-, and high-risk groups on the basis of the probably of recurrence from the hybrid model (Figure 8). Kaplan–Meier analysis demonstrated a highly significant overall therapeutic advantage for the combination approach (Figure 9A), with the combined TA + IPL group yielding a remarkably superior 12-month RFS rate compared to the TA-only and IPL-only cohorts (Log-rank *p* < 0.0001). When patients were stratified into sub-cohorts using the AI-enhanced hybrid prediction model (Figure 9B), the combined TA + IPL strategy consistently achieved the highest RFS across all risk tiers. This therapeutic superiority was highly significant in both the low-risk (*p* < 0.0001) and intermediate-risk (*p* = 0.0061) strata. Crucially, in the high-risk stratum comprising patients with the highest baseline disease burden, the combined TA + IPL approach still maintained the highest RFS trajectory, demonstrating a strong, clinically meaningful trend toward superior risk reduction despite the limited sample size (*p* = 0.059). To elucidate the “black-box” nature of the AI, SHAP analysis was implemented (Figure 10). Age and treatment frequency were identified as the most impactful features, with higher values correlating positively with increased recurrence risk—a finding consistent with the results of regression analysis.

### Guided intervention and subgroup analysis

**Figure 11:**
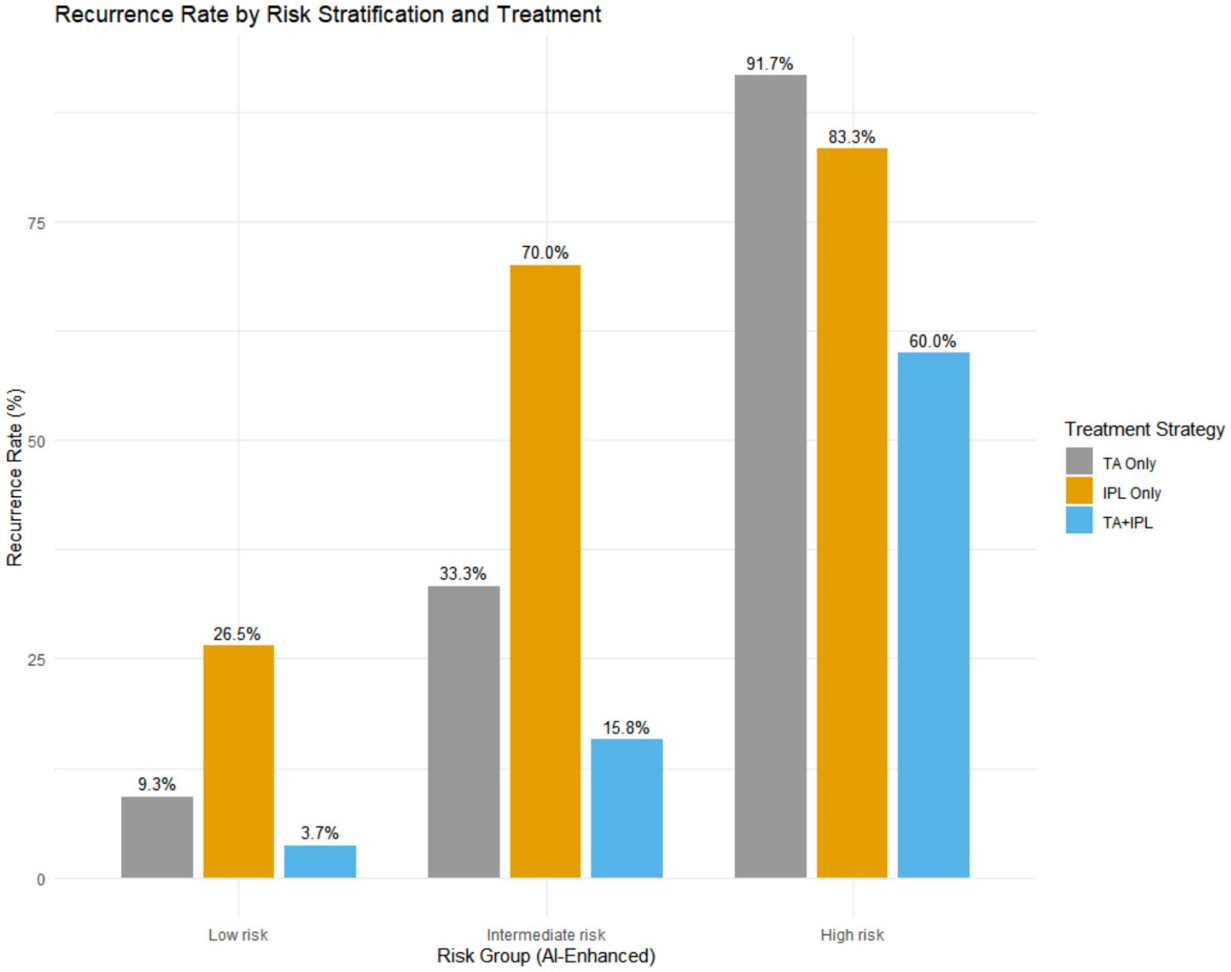
Comparison of recurrence rates across treatment modalities stratified by AI-enhanced risk. The bar chart illustrates recurrence rates across low-, intermediate-, and high-risk strata. Combined TA + IPL therapy (blue) consistently yields the lowest recurrence rates across all groups compared to TA-only (gray) and IPL-only (yellow) monotherapies. Notably, in the high-risk subgroup, the combined treatment reduces the recurrence rate to 60.0%, demonstrating a substantial protective effect relative to the high recurrence observed in the monotherapy cohorts (91.7 and 83.3%, respectively).

The practical utility of risk stratification was explored by comparing treatment outcomes within each risk stratum (Figure 11). In the low-risk group, recurrence rates remained relatively low across all modalities, with the TA + IPL combined therapy showing the lowest incidence (3.7%) compared to TA-only (9.3%) and IPL-only (26.5%). In the intermediate-risk group, the recurrence rates for monotherapies increased substantially (33.3% for TA-only and 70.0% for IPL-only), whereas the combined therapy continued to offer superior clinical control with a significantly lower recurrence rate of 15.8%. Most strikingly, in the high-risk group, recurrence rates escalated drastically for monotherapies, reaching 91.7% in the TA-only group and 83.3% in the IPL-only group. Although the TA + IPL combined therapy also saw an increase in this refractory population, it still maintained a markedly lower recurrence rate (60.0%) compared to either single treatment. These results clinically validate the ability of the hybrid model to effectively risk-stratify patients and demonstrate that the combined approach provides a consistent protective advantage, particularly as the baseline risk increases.

## Discussion

Chalazia often manifest as an insidious, painless growth that eludes early detection, making the precise onset of chalazion formation difficult to trace and clinical progression reliant solely on physical examination. In addition to local obstruction, the pathogenesis of chalazion is intertwined with a myriad of systemic and local risk factors, ranging from viral infection, *Demodex* infection, and blepharitis to systemic conditions such as rosacea, gastric disorders, and dyslipidemia (27, 28). Clinically, we observed that patients with recalcitrant or multiple chalazia experienced a marked decrease in both lesion count and recurrence frequency after receiving IPL combined with meibomian gland expression (MGX). This aligns with findings that antibiotic-augmented IPL therapy can effectively manage stubborn cases resistant to conventional surgical interventions (29). Nevertheless, the notably high 12-month recurrence rate observed in our standalone IPL cohort (38.2%) highlights critical mechanistic divergence among modalities. The resolution of a chalazion, a localized lipogranulomatous reaction secondary to ductal blockage, relies on the suppression of the established inflammatory nidus. Intralesional TA directly inhibits macrophage-mediated pathways, accelerating granuloma involution and minimizing the risk of recurrence driven by residual inflammatory tissue.

Evidence suggests that chalazion patients harbor significant ocular surface dysbiosis. Notably, the relative abundance of *Corynebacterium mastitidis*, a key player in local immune homeostasis, substantially decreased, whereas the abundance of *Staphylococcus* and *Acinetobacter* populations underwent pathological shifts (30). We hypothesize that IPL exerts its therapeutic effect primarily by modulating this microbial environment and targeting upstream causative mechanisms. Research has demonstrated that localized IPL irradiation significantly alleviates objective signs and symptoms, prolongs the TBUT, and restores gland function (31). Specifically, IPL shows superior efficacy in granulomatous-type chalazia than in cystic types of chalazia, achieving success rates comparable to those of surgery (32). By delivering skin-penetrating thermal energy, IPL selectively targets the meibomian glands, liquefying inspissated lipids and improving meibum characteristics—a cumulative effect evidenced by a reduced cyst diameter and an expanded functional gland area (33). However, since IPL lacks the ability of corticosteroids to potently and acutely suppress organized granulomatous tissue, incomplete initial resolution likely accounted for the greater recurrence observed in the monotherapy group during follow-up.

While a chalazion may appear as a focal lesion, its recurrence frequently indicates widespread MGD. Our multivariate analysis revealed MGD as the most robust independent predictor of recurrence (HR = 2.19), reinforcing the concept that recurrent focal lesions are localized manifestations of diffuse glandular impairment. Furthermore, the significant association between chronic (HR = 1.73) and recurrence suggests that prolonged exposure to inflammatory stimuli may induce fibrotic remodeling of the ductal epithelium. The role of *Demodex* infection also cannot be ignored; patients with *Demodex* infection exhibit significantly higher rates of chalazion recurrence (34), a trend that is particularly pronounced in pediatric cohorts (35). Our data confirm that demodex infection remains a critical determinant of therapeutic failure.

Consequently, for high-risk patients, focal lesion management is insufficient; systemic management of upstream mechanisms via tools such as IPL is imperative. The disparity in the recurrence rate—which was highest in the IPL-only group—stems from different targets within the inflammatory cascade. While TA induces rapid granuloma atrophy (median resolution: 14.1 days), IPL acts as a “microenvironmental stabilizer” by liquefying meibum and reducing the microbial load, albeit with a slower acute response time (32.5 days). Remarkably, the combined therapy cohort achieved near-instant resolution (7.6 days) and a minimal recurrence rate (8.3%). This synergy between immediate suppression and environmental repair represents a paradigm shift from simple lesion treatment to the restoration of global glandular homeostasis.

The superiority of this combined approach was particularly evident in patients identified as “high risk” by our hybrid AI model, which demonstrated robust predictive performance (C-index = 0.813). While low-risk patients fared well with conventional care, the practical utility of our model was most pronounced in the high-risk stratum typically characterized by severe MGD and chronic pathologies. In this cohort, combined TA + IPL therapy maintained a significantly lower recurrence rate (60.0%), a stark contrast to the high failure rates observed with monotherapies (91.7% for TA-only and 83.3% for IPL-only). This dramatic difference justifies a “mechanism-based, risk-stratified” management strategy. Our AI-enhanced model, validated through 1,000 bootstrap iterations (Brier score = 0.1012) and elucidated via SHAP analysis, transcends the limitations of traditional scoring systems. This model provides clinicians with an interpretable, data-driven roadmap to identify individuals who will derive the greatest benefit from early TA + IPL intervention, effectively transitioning clinical practice from empirical treatment to precision medicine.

Despite its rigor, this study is not without limitations. As a single-center observational study, potential selection bias may exist, although it was statistically mitigated through multivariate Cox regression. Future multicenter randomized controlled trials (RCTs) are warranted to confirm these causal effects. The study population exhibited a wide age range from 1 to 82 years, representing a highly heterogeneous cohort. Although this reflects the natural clinical spectrum of chalazion patients, the underlying etiology and compliance may differ significantly between pediatric and elderly populations. Additionally, while the 12-month follow-up captures most recurrences, it may not fully reflect the long-term structural remodeling benefits of IPL; subsequent studies should extend observations to 24–36 months. Integration of biomarkers, such as tear film cytokines or infrared meibography, could further refine the model’s predictive accuracy. Finally, the generalizability of the AI algorithm across diverse ethnicities or systemic disease profiles requires external validation in independent global datasets.

## Conclusion

In summary, this study demonstrates that risk-based stratification effectively identifies chalazion patients at varying risk of recurrence. Combined TA + IPL therapy offers profound clinical benefits, particularly for high-risk individuals. The nomogram and AI-enhanced hybrid framework developed herein provide a robust, interpretable tool for individualized treatment planning. By merging traditional statistics with machine learning and SHAP-based interpretability, we offer a scientific foundation for implementing precision medicine in chalazion management, optimizing patient outcomes through targeted intervention.

## Data Availability

The original contributions presented in the study are included in the article/supplementary material, further inquiries can be directed to the corresponding authors.
